# BioTorrents: A File Sharing Service for Scientific Data

**DOI:** 10.1371/journal.pone.0010071

**Published:** 2010-04-14

**Authors:** Morgan G. I. Langille, Jonathan A. Eisen

**Affiliations:** Genome Center, University of California Davis, Davis, California, United States of America; University of California Riverside, United States of America

## Abstract

The transfer of scientific data has emerged as a significant challenge, as datasets continue to grow in size and demand for open access sharing increases. Current methods for file transfer do not scale well for large files and can cause long transfer times. In this study we present BioTorrents, a website that allows open access sharing of scientific data and uses the popular BitTorrent peer-to-peer file sharing technology. BioTorrents allows files to be transferred rapidly due to the sharing of bandwidth across multiple institutions and provides more reliable file transfers due to the built-in error checking of the file sharing technology. BioTorrents contains multiple features, including keyword searching, category browsing, RSS feeds, torrent comments, and a discussion forum. BioTorrents is available at http://www.biotorrents.net.

## Introduction

**Figure 1:**
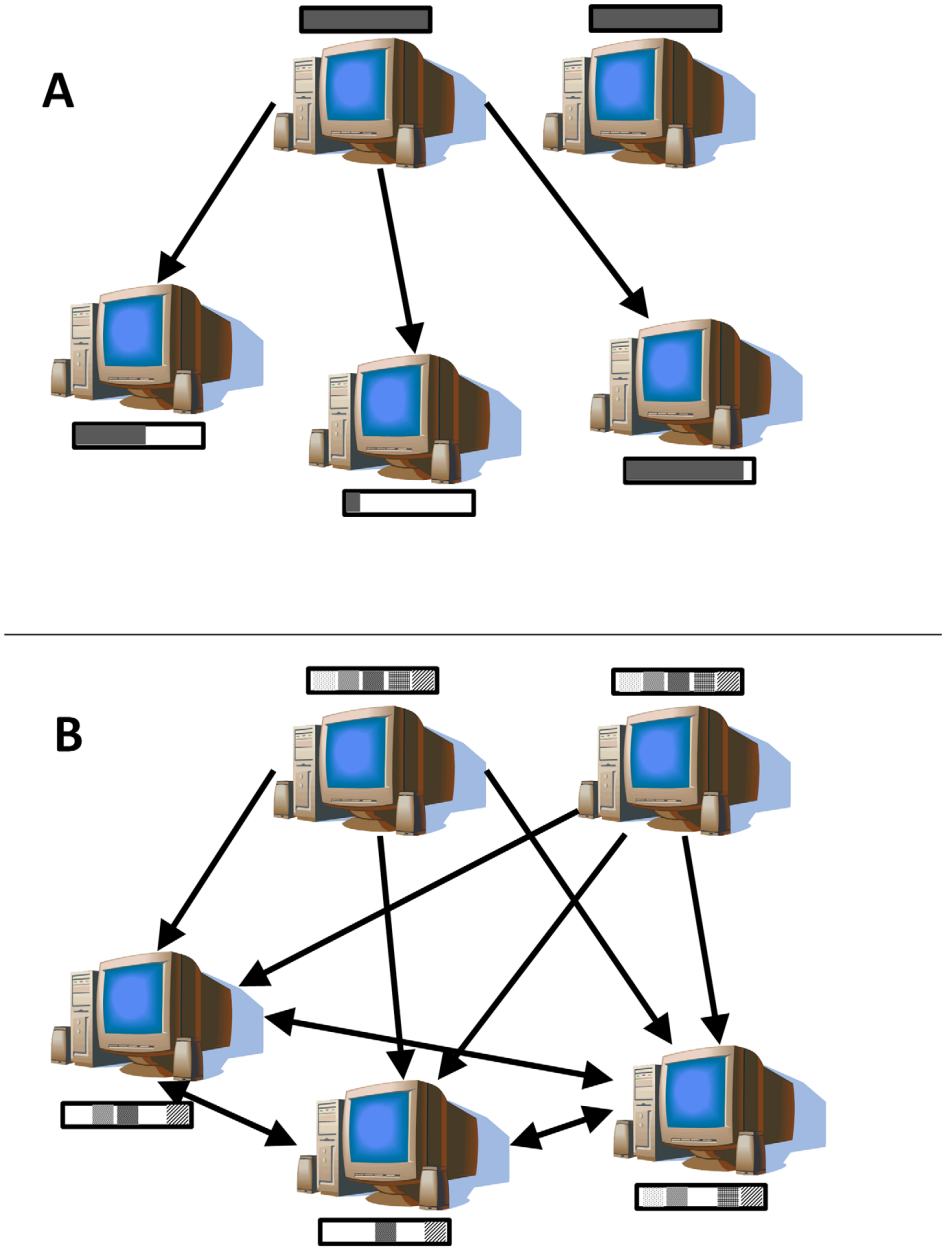
Illustration of differences between traditional and peer to peer file transfer protocols. A) Traditional file transfer protocols such as HTTP and FTP use a single host for obtaining a dataset (grey filled black box), even though other computers contain the same file or partial copies while downloading (partially filled black box). This can cause transfers to be slow due to bandwidth limitations or if the host fails. B) The peer-to-peer file transfer protocol, BitTorrent, breaks up the dataset into small pieces (shown as pattern blocks within black box), and allows sharing among computers with full copies or partial copies of the dataset. This allows faster transfer times and decentralization of the data.

The amount of data being produced in the sciences continues to expand at a tremendous rate[1]. In parallel, and also at an increasing rate, is the demand to make this data openly available to other researchers, both pre-publication[2] and post-publication[3]. Considerable effort and attention has been given to improving the portability of data by developing data format standards[4], minimal information for experiment reporting[5]–[8], data sharing polices[9], and data management[10]–[13]. However, the practical aspect of moving data from one location to another has relatively stayed the same; that being the use of Hypertext Transfer Protocol (HTTP) [14] or File Transfer Protocol (FTP) [15]. These protocols require that a single server be the source of the data and that all requests for data be handled from that single location (Fig. 1A). In addition, the server of the data has to have a large amount of bandwidth to provide adequate download speeds for all data requests. Unfortunately, as the number of requests for data increases and the provider's bandwidth becomes saturated, the access time for each data request can increase rapidly. Even if bandwidth limitations are very large, these file transfer methods require that the data is centrally stored, making the data inaccessible if the server malfunctions.

Many different solutions have been proposed to help with many of the challenges of moving large amounts of data. Bio-Mirror (http://www.bio-mirror.net/) was started in 1999 and consists of several servers sharing the same identical datasets in various countries. Bio-mirror improves on download speeds, but requires that the data be replicated across all servers, is restricted to only very popular genomic datasets, and does not include the fast growing datasets such as the Sequence Read Archive (SRA) (http://www.ncbi.nlm.nih.gov/sra). The Tranche Project (https://trancheproject.org/) is the software behind the Proteome Commons (https://proteomecommons.org/) proteomics repository. The focus of the Tranche Project is to provide a secure repository that can be shared across multiple servers. Considering that all bandwidth is provided by these dedicated Tranche servers, considerable administration and funding is necessary in order to maintain such a service. An alternative to these repository-like resources is to use a peer-to-peer file transfer protocol. These peer-to-peer networks allow the sharing of datasets directly with each other without the need for a central repository to provide the data hosting or bandwidth for downloading. One of the earliest and most popular peer-to-peer protocols is Gnutella (http://rfc-gnutella.sourceforge.net/) which is the protocol behind many popular file sharing clients such as LimeWire (http://www.limewire.com/), Shareaza (http://shareaza.sourceforge.net/), and BearShare (http://www.bearshare.com/). Unfortunately, this protocol was centered on sharing individual files and does scale well for sharing very large files. In comparison, the BitTorrent protocol [16] handles large files very well, is actively being developed, and is a very popular method for data transfer. For example, BitTorrent can be used to transfer data from the Amazon Simple Storage Service (S3) (http://aws.amazon.com/s3/), is used by Twitter (http://twitter.com/) as a method to distribute files to a large number of servers (http://github.com/lg/murder), and for distributing numerous types of media.

The BitTorrent protocol works by first splitting the data into small pieces (usually 514 Kb to 2 Mb in size), allowing the large dataset to be distributed in pieces and downloaded from various sources (Fig. 1B). A checksum is created for each file piece to verify the integrity of the data being received and these are stored within a small “torrent” file. The torrent file also contains the address of one or more “trackers”. The tracker is responsible for maintaining a list of clients that are currently sharing the torrent, so that clients can make direct connections with other clients to obtain the data. A BitTorrent software client (see Table 1) uses the data in the torrent file to contact the tracker and allow transferring of the data between computers containing either full or partial copies of the dataset. Therefore, bandwidth is shared and distributed among all computers in the transaction instead of a single source providing all of the required bandwidth. The sum of available bandwidth grows as the number of file transfers increases, and thus scales indefinitely. The end result is faster transfer times, less bandwidth requirements from a single source, and decentralization of the data.

**Table 1:** Comparison of several popular BitTorrent software clients and their features.

BitTorrent Client Name	Operating System^1^			Interface^2^			RSS^3^	LPD^4^	DHT^5^
	Win.	Mac.	Linux	GUI	Web	CLI			
uTorrent	X	X		X	X		X	X	X
Deluge	X	X	X	X	X			X	X
Vuze	X	X	X	X	X		X		X
Transmission		X	X	X	X	X			X
rTorrent		X	X			X			X
kTorrent	X	X	X	X	X		X		X

1 Win:Microsoft Windows, Mac: Mac OSX.

2 GUI: Graphical User Interface, Web: built-in web server interface, CLI: command line interface.

3 RSS download can be obtained for all clients by using RSSDler (http://code.google.com/p/rssdler/).

4 LPD: Local Peer Discovery.

5 DHT: Distributed Hash Table.

Torrent files have been hosted on numerous websites and in theory scientific data can be currently transferred using any one of these BitTorrent trackers. However, many of these websites contain materials that violate copyright laws and are prone to being shut down due to copyright infringement. In addition, the vast majority of data on these trackers is non-science related and makes searching or browsing for legitimate scientific data nearly impossible. Therefore, to improve upon the open sharing of scientific data we created BioTorrents, a legal BitTorrent tracker that hosts scientific data and software.

## Results

### Tracker and Reliability of Service

The most basic requirement of any torrent server software is the actual “tracker” that individual torrent clients interact with to obtain information about where to download pieces of data for a particular torrent. In order to minimize any possible transfer disruptions arising from the BioTorrents tracker not being accessible, a secondary tracker is added automatically to all new torrents uploaded to BioTorrents. Currently this backup tracker is set to use the Open BitTorrent Tracker (http://openbittorrent.com/). Also, many BitTorrent clients support a distributed hash table (DHT) for peer discovery, which often allows data transfer to continue in the absence of a tracker, further enhancing the reliability over traditional client-server file transfers.

### Obtaining Data

In addition to the basic tracker, BioTorrents has several features supporting the finding, sharing, and commenting of torrents. Relevant torrents can be found by browsing categories (genomics, transcriptomics, papers, etc.), license types (Public Domain, Creative Commons, GNU General Public License, etc.) and by using the provided text search. Also, torrents are indexed by Google (http://www.google.com) allowing users searching for datasets, but unaware of BioTorrents existence, to be directed to their availability on BioTorrents. Information about each dataset on BioTorrents is supplied on a details page giving a description of the data, number of files, date added, user name of the person who created the dataset, and various other details including a link to the actual torrent file. To begin downloading of a dataset, the user downloads and opens the torrent file in the user's previously installed BitTorrent client software (Table 1). The user can then control many aspects of their download (stopping, starting, download limits, etc.) through their client software without any further need to visit the BioTorrents webpage. The BitTorrent client will automatically connect with other clients sharing the same torrent and begin to download pieces in a non-random order. The integrity of each data piece is verified using the original file hash provided in the downloaded torrent ensuring that the completed download is an exact copy. The BitTorrent client contacts the BioTorrents tracker frequently (approximately every 30 minutes) to obtain the addresses of other clients and also to report statistics of how much data they have downloaded and uploaded. These statistics are linked to the user's profile (default is the guest account), to allow real-time display on BioTorrents of who is sharing a particular dataset.

The choice of BitTorrent client will depend on the operating system and options that the user requires. For example, some BitTorrent clients (see Table 1) have a feature called Local Peer Discovery (LPD), that searches for other computers sharing the same data on their local area network (LAN), and allows rapid direct transfer of data over the shared network instead of over the internet. This situation may arise often in research institutions where LANs are often quite large and multiple researchers are working on similar datasets. Another significant feature of the BitTorrent client, uTorrent, is the addition of a newly designed transfer protocol called uTP[17], that is able to monitor and adapt to network congestion by limiting its transfer speeds when other network traffic is detected. This functionality is important for system administrators and internet service providers (ISPs) that may have previously attempted to block or hinder BitTorrent activity due its bandwidth saturating effects.

### Sharing Data

Sharing data on BioTorrents is a simple three step process. First, the user creates a torrent file on their personal computer using the same BitTorrent client software that is used for downloading (Table 1). The only piece of information the user needs to create the torrent, is the BioTorrents tracker announce URL which is personalized for each user (see below), and is located on the BioTorrents upload page. Second, this newly created torrent file is uploaded on the “BioTorrents - Upload” page along with a user description, category, and license type for the data. Third, the user leaves their computer/server on with their BitTorrent client running so that other users can download the data from them.

It should be noted that only users that have created a free account with BioTorrents are able to upload new torrents. This is to limit any possible spamming of the website as well as provide accountability for the data being shared. BioTorrents enforces this and tracks users by giving each user a passkey. This passkey is automatically embedded within each torrent file that is downloaded from BioTorrents and is appended to the BioTorrents tracker's announce URL. Although, we would hope that most users create an account on BioTorrents, we still allow anyone to download torrents without doing so.

An alternative upload method is provided for more advanced users that have many datasets to share and/or are sharing data from a remote Linux based server. This method uses a Perl (http://www.perl.org) script that takes the dataset to be shared as input and returns a link to the dataset on BioTorrents along with the torrent file; therefore, allowing torrents to be created for numerous datasets automatically. This feature would be useful for institutions or data providers that would like to add a BitTorrent download option for their datasets.

Considering that many datasets in science are often updated, BioTorrents allows torrents to be optionally grouped into versions. This functionality allows improved browsing of BioTorrents by providing links between torrents. More importantly, this versioning classification allows users interested in certain software or datasets to be notified via a Really Simple Syndication (RSS) feed that a new version is available on BioTorrents. In addition, this RSS feed can be used to obtain automated updates for datasets that are often changing, such as genomic and protein databases. For example, a user could copy the RSS feed for a dataset that is being updated often on BioTorrents (weekly, monthly, etc.) into their BitTorrent RSS capable client. When a new version is released on BioTorrents the BitTorrent client automatically downloads the torrent file, checks to see what parts of the data have changed, and downloads only pieces that have been updated.

The speed and effectiveness of the BitTorrent protocol depends on the number of peers; in particular, those peers that have a complete copy of the file and can act as “seeds”. Therefore, it is important that individuals or institutions act as seeds to achieve full potential. Currently, all newly added data is automatically downloaded and shared from the BioTorrents server. This is to ensure that each dataset always has at least one server available for downloading. As the number of datasets and users of BioTorrents increases, and to improve on transfer speeds on a geospatial scale (i.e. across countries and continents), we would encourage other institutions to automatically download and share all or some of the data on BioTorrents.

### Discussion Forum, Comments, RSS, and FAQ

Any logged in BioTorrents user can write comments or questions about a particular torrent directly on its details page. This can provide useful feedback both to the creator of the dataset as well as to other users downloading it. Alternatively, researchers wanting to discuss more general questions about BioTorrents, particular datasets, or science, can use the provided “BioTorrents - Forums”. Comments and discussion posts can be read by all visitors, but a free account is necessary to post to either of these. Users that would like to be updated on newly uploaded datasets can use the BioTorrents RSS web feed. The RSS feeds can be configured for certain categories, license types, users, and search terms, and can also be used with many BitTorrent clients to automatically download all or some of the datasets on BioTorrents without human intervention. Finally, the “BioTorrents – FAQ” (Frequently Asked Questions) page provides users with information about BitTorrent technology and general help for using BioTorrents for both downloading and sharing of data.

## Discussion

BitTorrent technology can supplement and extend current methods for transferring and publishing of scientific data on various scales. Large institutions and data repositories such as GenBank[18], could offer their popular or larger datasets via BioTorrents as an alternative method for download with minimal effort. The amount of data being transferred by these large institutions should not be underestimated. For example, in a single month NCBI users downloaded the 1000 Genomes (8981 GB), Bacteria Genomes (52 GB), Taxonomy (1GB), GenBank (233 GB), and Blast Non-Redundant (NR) (3 GB) datasets; 100000, 30000, 15000, 10000, and 7000 times, respectively (personal correspondence). If BitTorrent technology was implemented for these datasets then the data supplier would benefit from decreased bandwidth use, while researchers downloading the data, especially those not on the same continent as the data supplier, enjoy faster transfer times.

Small groups or individual researchers can also benefit from using BioTorrents as their primary method for publishing data. Although, these less popular datasets may not enjoy the same speed benefits from using the BitTorrent protocol due to the lack of data exchange among simultaneous downloads, the lower barrier of entry to providing data compared with running a personal web server, and the ability to operate behind routers employing network address translation (NAT) makes the use of BioTorrents for less popular datasets still beneficial. In addition, BioTorrents allows researchers to make their data, software, and analyses available instantly, without the requirement of an official submission process or accompanying manuscript. This form of data publishing allows open and rapid access to information that would expedite science, especially for time-sensitive events such as the recent outbreaks of influenza H1N1[19] or severe acute respiratory syndrome (SARS)[20]. No matter what the circumstance, BioTorrents provides a useful resource for advancing the sharing of open scientific information.

### Implementation

The source code for BioTorrents.net was derived from the TBDev.net (http://tbdev.net) GNU General Public Licensed (GPL) project. The dynamic web pages are coded in PHP with some features being implemented with JavaScript. All information, including information about users, torrents, and discussion forums are stored in a MySQL database. The original source code was altered in various ways to allow easier use of BioTorrents by scientists; the most significant being, that anyone can download torrents without signing up for an account. In addition, torrents can be classified by various categories and license types, and grouped with other alternative versions of torrents.

### Availability

The BioTorrents web server along with the source code is available freely under the GNU General Public License at http://www.biotorrents.net.
